# A new method based on the manifold-alternative approximating for low-rank matrix completion

**DOI:** 10.1186/s13660-018-1931-4

**Published:** 2018-12-11

**Authors:** Fujiao Ren, Ruiping Wen

**Affiliations:** 1 Department of Mathematics, Taiyuan Normal University, Shanxi, P.R. China; 2 Key Laboratory of Engineering & Computing Science, Shanxi Provincial Department of Education/Department of Mathematics, Taiyuan Normal University, Shanxi, P.R. China

**Keywords:** Manifold-alternative approximating, Low rank, Matrix completion, Convergence

## Abstract

In this paper, a new method is proposed for low-rank matrix completion which is based on the least squares approximating to the known elements in the manifold formed by the singular vectors of the partial singular value decomposition alternatively. The method can achieve a reduction of the rank of the manifold by gradually reducing the number of the singular value of the thresholding and get the optimal low-rank matrix. It is proven that the manifold-alternative approximating method is convergent under some conditions. Furthermore, compared with the augmented Lagrange multiplier and the orthogonal rank-one matrix pursuit algorithms by random experiments, it is more effective as regards the CPU time and the low-rank property.

## Introduction

Matrix completion, proposed by Candès and Recht [7] in 2009, is a challenging problem. There has been a lot of study (see [1–8, 11–19, 23–28, 30, 33–35]) both in theoretical and algorithmic aspects on this problem. Explicitly seeking the lowest-rank matrix consistent with the known entries is mathematically expressed as

1.1 $$\begin{aligned} &\min_{X\in\mathbb{R}^{n\times n}} \operatorname{rank}(X) \\ & \quad \mbox{subject to } X_{ij}=M_{ij},\quad (i,j)\in \varOmega, \end{aligned}$$

where the matrix $M\in\mathbb{R}^{n\times n}$ is the unknown matrix, *Ω* is a random subset of indices for the known entries. The problem occurs in many areas of engineering and applied science, such as model reduction [20], machine learning [1, 2], control [22], pattern recognition [10], imaging inpainting [3] and computer vision [29].

As is well known, Candés and Rechat [7] replaced the rank objective in (1.1) with its convex relaxation, and they showed that the lowest-rank matrices could be recovered exactly from most sufficiently large sets of sampled entries by computing the matrix of minimum nuclear norm that agreed with the provided entries, i.e., the exact matrix completion via convex optimization, as follows:

1.2 $$\begin{aligned} &\min_{X\in\mathbb{R}^{n\times n}} \Vert X \Vert _{*} \\ &\quad \mbox{subject to } X_{ij}=M_{ij},\quad (i,j)\in \varOmega, \end{aligned}$$

where the functional $\|X\|_{*}$ is the nuclear norm of the matrix *X*, the unknown matrix $M\in\mathbb{R}^{n\times n}$ of *r*-rank is square, and one has available *m* sampled entries $\{M_{ij}:(i,j)\in\varOmega\}$ with *Ω* a random subset of cardinality *m*.

There have been many algorithms which were designed to attempt to solve the global minimum of (1.2) directly. For example, the hard thresholding algorithms [4, 15, 17, 26], the singular value theresholding (SVT) method [6], the accelerated singular values thresholding method (ASVT [14]), the proximal forward–backward splitting [9], the augmented Lagrange multiplier (ALM [19]) method, the interior point methods [7, 28], and the new gradient projection (NGP [34]) method.

Based on the bi-linear decomposition of an *r*-rank matrix, some algorithms have been presented to solve (1.1) under the *r*-rank that is known or can be estimated [20, 21]. We mention the Riemannian geometry method [30] and the Riemannian trust-region method [5, 23], the alternating minimization method [16] and the alternating steepest descent method [26]. The rank of many completion matrices, however, is unknown, so that one has to estimate it ahead of time or approximate it from a lower rank, which causes the difficulty of solving the matrix completion problem. Wen et al. [33] presented the two-stage iteration algorithms for the unknown-rank problem. To decrease the computational cost, based on extending the orthogonal matching pursuit (OMP) procedure from the vector to matrix level, Wang et al. [31] presented an orthogonal rank-one matrix pursuit (OR1MP) method, in which only the top singular vector pair was calculated at each iteration step and an *ϵ*-feasible solution can be obtained in only $O(\log(\frac{1}{\epsilon}))$ iterations with less computational cost. However, the method converges to a feasible point rather than the optimal one with minimization rank such that the accuracy is poor and cannot be improved if the rank is reached. In this study, we come up with a manifold-alternative approximating method for solving the problem (1.2) motivated by the above. In an outer iteration, the approximated process can be done in the left-singular vector subspace and the approximation will be alternatively carried out in the right-singular vector subspace in an inner iteration. In a whole iteration, the reduction of the rank results in an alternating optimization, while the completed matrix satisfies $M_{ij}=(UV^{T})_{ij}$, for $(i,j)\in\varOmega$.

Here are some notations and preliminaries. Let $\varOmega\subset\{ 1,2,\ldots,n\}\times\{1,2,\ldots,n\}$ denote the indices of the observed entries of the matrix $X\in\mathbb{R}^{n\times n}$, *Ω̄* denote the indices of the missing entries. $\|X\|_{*}$ represents the nuclear norm (also called Schatten 1-norm) of *X*, that is, the sum of the singular values of *X*, $\|X\|_{2}, \|X\|_{F}$ denote 2-norm and *F*-norm of *X*, respectively. We denote by $\langle X,Y\rangle=\operatorname{trace}(X^{*},Y)$ the inner product between two matrices $(\|X\|_{F}^{2}=\langle X,X\rangle)$. The Cauchy–Schwartz inequality gives $\langle X,Y\rangle\leq\|X\|_{F}\cdot\|Y\|_{F}$ and it is well known that $\langle X,Y\rangle\leq\|X\|_{2}\cdot\|Y\|_{*}$ [7, 32].

For a matrix $A\in\mathbb{R}^{n\times n}$, $\operatorname{vec}(A)=(a_{1}^{T},a_{2}^{T},\ldots ,a_{n}^{T})^{T}$ denotes a vector reshaped from matrix *A* by concatenating all its column vectors, dim$(A)$ is always used to represent the dimensions of *A* and $r(A)$ stands for the rank of *A*.

The rest of the paper is organized as follows. After we provide a brief review of the ALM and the OR1MP methods, a manifold-alternative approximating method is proposed in Sect. 2. The convergence results of the new method are discussed in Sect. 3. Finally, numerical experiments are shown with comparison to other methods in Sect. 4. We end the paper with a concluding remark in Sect. 5.

## Methods

### The method of augmented Lagrange multipliers

The method of augmented Lagrange multipliers (ALMs) was proposed in [19] for solving a convex optimization (1.2). It should be described subsequently.

Since the matrix completion problem is closely connected to the robust principal component analysis (RPCA) problem, it can be formulated in the same way as RPCA, an equivalent problem of (1.2) can be considered as follows.

As *E* will compensate for the unknown entries of *M*, the unknown entries of *M* are simply set as zeros. Suppose that the given data are arranged as the columns of a large matrix $M\in\mathbb{R}^{m\times n}$. The mathematical model for estimating the low-dimensional subspace is to find a low-rank matrix $X\in\mathbb{R}^{m\times n}$, such that the discrepancy between *X* and *M* is minimized, leading to the following constrained optimization:

2.1 $$\begin{aligned} &\min_{X,E\in\mathbb{R}^{m\times n}} \Vert X \Vert _{*} \\ &\quad \mbox{subject to } X+E=M,\quad \pi_{\varOmega}(E)=0, \end{aligned}$$

where $\pi_{\varOmega}: \mathbb{R}^{m\times n}\rightarrow\mathbb{R}^{m\times n}$ is a linear operator that keeps the entries in *Ω* unchanged and sets those outside *Ω* (say, in *Ω̅*) zeros. Then the partial augmented Lagrange function is

$$L(X,E,Y,\mu)= \Vert X \Vert _{*}+\langle Y, M-X-E\rangle+\frac{\mu}{2} \Vert M-X-E \Vert _{F}^{2}. $$

The augmented Lagrange multipliers method is summarized in the following:

#### Method 2.1

(Algorithm 6 of [19])

**Input:** Observation samples $M_{ij}, (i,j)\in\varOmega$, of matrix $M\in\mathbb{R}^{m\times n}$.

1. $Y_{0}=0$; $E_{0}=0$; $\mu_{0}>0$; $\rho>1$; $k=0$.
2. while not converged do
3. // Lines 4–5 solve $A_{k+1}=\arg\min_{X} L(X,E_{k},Y_{k},\mu_{k})$.
4. $(U,S,V)=\operatorname{svd}(M-E_{k}-\mu_{k}^{-1}Y_{k})$;
5. $A_{k+1}=US_{\mu_{k}^{-1}}[S]V^{T}$.
6. // Line 7 solves $E_{k+1}=\arg\min_{\pi_{\varOmega}(E)=0}L(A_{k+1},E,Y_{k},\mu_{k})$.
7. $E_{k+1}=\pi_{\overline{\varOmega}}(M-X_{k+1}+\mu_{k}^{-1}Y_{k})$.
8. $Y_{k+1}=Y_{k}+\mu_{k}(M-X_{k+1}-E_{k+1})$.
9. Update $\mu_{k}$ to $\mu_{k+1}$.
10. $k\leftarrow k+1$.
11. end while

**Output:** $(X_{k},E_{k})$.

#### Remark

It is reported that the method of augmented Lagrange multipliers has been applied to the problem (1.2). It is of much better numerical behavior, and it is also of much higher accuracy. However, the method has the disadvantage of the penalty function: the matrix sequences $\{X_{k}\}$ generated by the method are not feasible. Hence, the accepted solutions are not feasible.

### The method of the orthogonal rank-one matrix pursuit (OR1MP)

We proceed based on the expression of the matrix $X\in\mathbb {R}^{m\times n}$,

2.2 $$\begin{aligned} X=M(\theta)=\sum_{i\in\varLambda} \theta_{i}M_{i}, \end{aligned}$$

where $\{M_{i}: i\in\varLambda\}$ is the set of all $m\times n$ rank-one matrices with unit Frobenius norm.

The original low-rank matrix approximation problem aims to minimize the zero-norm of the vector $\theta=(\theta_{i})_{i\in\varLambda}$ subject to the equality constraint

2.3 $$\begin{aligned} &\min_{\theta} \Vert \theta \Vert _{0} \\ &\quad \mbox{subject to } P_{\varOmega}\bigl(M(\theta)\bigr)=P_{\varOmega}(Y), \end{aligned}$$

where $\|\theta\|_{0}$ represents the number of nonzero elements of the vector *θ*, and $P_{\varOmega}$ is the orthogonal projector onto the span of matrices vanishing outside of *Ω*.

The authors in [31] reformulate further the problem as

2.4 $$\begin{aligned} &\min \bigl\Vert P_{\varOmega}\bigl(M(\theta) \bigr)-P_{\varOmega}(Y) \bigr\Vert _{F}^{2} \\ &\quad \mbox{subject to } \Vert \theta \Vert _{0}\leq r, \end{aligned}$$

they could solve it by an orthogonal matching pursuit (OMP) type algorithm using rank-one matrices as the basis. It is implemented by two steps alternatively: one is to pursue the basis $M_{k}$, and the other is to learn the weight of the basis $\theta_{k}$.

#### Method 2.2

(Algorithm 1 of [31])

**Input:** $Y_{\varOmega}$ and stopping criterion.

**Initialize:** Set $X_{0}=0; \theta^{0}=0$ and $k=1$.

**repeat**

*Step 1*: Find a pair of top left- and right-singular vectors $(u_{k},v_{k})$ of the observed residual matrix $R_{k}=Y_{\varOmega}-X_{k-1}$ and set $M_{k}=u_{k}v_{k}^{T}$.
*Step 2*: Compute the weight vector $\theta^{k}$ using the closed form least squares solution $\theta^{k}=(\bar{M}_{k}^{T}\bar {M}_{k})^{-1}\bar{M}_{k}^{T}\dot{y}$.
*Step 3*: Set $X_{k}=\sum_{i=1}^{k}\theta _{i}^{k}(M_{i})_{\varOmega}$ and $k\leftarrow k+1$.

**until** stopping criterion is satisfied

**Output:** Constructed matrix $\hat{Y}=\sum_{i=1}^{k}\theta_{i}^{k}M_{i}$.

#### Remark

To decrease the computational cost, based on extending the orthogonal matching pursuit (OMP) procedure from the vector to matrix level, Wang et al. [31] presented an orthogonal rank-one matrix pursuit (OR1MP) method, in which only the top singular vector pair was calculated at each iteration step and an *ϵ*-feasible solution can be obtained in only $O(\log(\frac{1}{\epsilon }))$ iterations with less computational cost. However, the method converges to a feasible point rather than the optimal one with minimization rank such that the accuracy is poor and cannot be improved if the rank is reached.

### The method of a manifold-alternative approximating (MAA)

For convenience, $[U_{k},\varSigma_{k},V_{k}]_{\tau_{k}}=\operatorname{lansvd}(Y_{k})$ denotes the top-$\tau_{k}$ singular pairs of the matrix $Y_{k}$ by using the Lanczos method, where $U_{k}=(u_{1},u_{2},\ldots,u_{\tau_{k}}), V_{k}=(v_{1},v_{2},\ldots,v_{\tau_{k}})$ and $\varSigma_{k}=\operatorname{diag}(\sigma _{1k},\sigma_{2k},\ldots,\sigma_{\tau_{k},k}), \sigma_{1k}\geq\sigma _{2k}\geq\cdots\geq\sigma_{\tau_{k},k}>0$.

Let

$$\mathcal{M}_{k}=\bigl\{ X\in\mathbb{R}^{n\times m}: \operatorname{rank}(X)=k\bigr\} $$

denote the manifold of fixed-rank matrices. Using the SVD, one has the equivalent characterization

2.5 $$\begin{aligned} \mathcal{M}_{k}=\bigl\{ U\varSigma V^{T}:U\in St_{k}^{m},V\in St_{k}^{n}, \varSigma= \operatorname{diag}(\sigma_{i}),\sigma_{1}\geq\cdots\geq \sigma_{k}>0\bigr\} , \end{aligned}$$

where $St_{k}^{m}$ is the Stiefel manifold of $m\times k$ real, orthogonal matrices, and $\operatorname{diag}(\sigma_{i})$ denotes a diagonal matrix with $\sigma_{i}$, $i=1,2 ,\ldots, k$ on the diagonal.

#### Method 2.3

(MAA)

**Input:** $D=P_{\varOmega}(M)$, $\operatorname{vec}(D)=D(i,j)$, $(i,j)\in\varOmega$, $\tau_{0}>0$ ($\tau_{k}\in{N}^{+}$), $0< c_{1},c_{2}<1$, a tolerance $\epsilon>0$.

**Initialize:** Set $Y_{0}=D$ and $k=0$.

**repeat**

*Step 1*: Compute the partial SVD of the matrix $Y_{k}: [U_{k},\varSigma_{k},V_{k}]_{\tau_{k}}=\operatorname{lansvd}(Y_{k})$.
*Step 2*: Solve the following optimization models, $\min\|\operatorname{vec}(D)-\operatorname{vec}(P_{\varOmega}(U_{k}X_{k}))\|_{F}$, set $Y_{k+1}=U_{k}X_{k}$.
*Step 3*: When $\frac{\|Y_{k+1}-Y_{k}\|_{F}}{\|D\| _{F}}<\epsilon$, stop; otherwise, go to the next step.
*Step 4*: For $k>0$, if $\|\operatorname{vec}(D)-\operatorname{vec}(P_{\varOmega}(Y_{k+1}))\|_{F}< c_{2}\|\operatorname{vec}(D)-\operatorname{vec}(P_{\varOmega}(Y_{k}))\|_{F}, \tau_{k+1}=[c_{1}\tau_{k}]$ go to the next step; otherwise, do
  1. Set $Z_{k}=D+P_{\overline{\varOmega}}(Y_{k+1})$, compute the partial SVD of the matrix $Z_{k}$: $[U_{k},\varSigma_{k},V_{k}]_{\tau_{k}}=\operatorname{lansvd}(Z_{k})$. Let $W_{K}=U_{k}\varSigma_{k}V_{k}^{T}, \alpha_{k}=\|\operatorname{vec}(D)-\operatorname{vec}(P_{\varOmega}(W_{k}))\|_{F}$.
    Set $Z_{k+\frac{1}{2}}=D+P_{\overline{\varOmega}}(W_{k})$.
  2. Do SVD:
    $$[U_{k+\frac{1}{2}},\varSigma_{k+\frac{1}{2}},V_{k+\frac{1}{2}}]_{\tau_{k}}= \operatorname{lansvd}(Z_{k+\frac{1}{2}}). $$
    Then $W_{k+\frac{1}{2}}=U_{k+\frac{1}{2}}\varSigma_{k+\frac{1}{2}}V_{k+\frac{1}{2}}^{T}$.
  3. Solve the following minimum problems, yielding $Y_{k+\frac{1}{2}}$ and $\alpha_{k+\frac{1}{2}}$, $\min \|\operatorname{vec}(D)-\operatorname{vec}(P_{\varOmega}(X_{k+\frac {1}{2}}V_{k+\frac{1}{2}}^{T}))\|_{F}$, set $Y_{k+\frac{1}{2}}=X_{k+\frac{1}{2}}V_{k+\frac{1}{2}}^{T}$, $\alpha_{k+\frac {1}{2}}=\|\operatorname{vec}(D)-\operatorname{vec}(P_{\varOmega}(Y_{k+\frac{1}{2}}))\| _{F}$.
    Set $Z_{k+1}=D+P_{\overline{\varOmega}}(Y_{k+\frac{1}{2}})$.
  4. If $\alpha_{k+\frac{1}{2}}\leq c_{2}\alpha_{k}$, $\tau_{k+1}=\tau_{k}-1$; if $\alpha_{k+\frac{1}{2}}\geq\alpha_{k}$, $\tau_{k+1}=\tau_{k}+1$, go to Step 1. Otherwise, if $c_{2}\alpha_{k}\leq \alpha_{k+\frac{1}{2}}<\alpha_{k}$, $\tau_{k+1}=\tau_{k}$, go to the next step.
*Step 5*: $k:=k+1$, go to Step 2.

**until** stopping criterion is satisfied

**Output:** Constructed matrix $Y_{k}$.

## Convergence analysis

Now, the convergence theory will be discussed in the following.

### Lemma 3.1

*Let* $Y^{*}$ *be the optimal solution of* (1.1). *Then there exists a nonnegative number* $\varepsilon_{0}$ *such that*

$$\bigl\Vert Y-Y^{*} \bigr\Vert _{F}\geqslant\varepsilon_{0} $$

*if and only if for any matrix* *Y*, $r(Y)< r(Y^{*})$.

### Proof

From the discretional nature of the rank, there exists $Y_{\varepsilon}$ that satisfies

$$r(Y_{\varepsilon})\leqslant r(Y)-1, \quad \forall\varepsilon>0, $$

and

$$\bigl\Vert Y_{\varepsilon}-Y^{*} \bigr\Vert _{F}< \varepsilon. $$

Hence, $Y_{\varepsilon}\rightarrow Y_{*}$ if $\varepsilon\rightarrow0$.

This is in contrast to $r(Y^{*})\leqslant r(Y^{*})-1$. □

### Lemma 3.2

*Assume that the manifolds* $W_{k+\frac{1}{2}}$, $W_{k}$ *satisfy*

$$r(W_{k+\frac{1}{2}})\geq r(W_{k}), $$

*then*

$$\alpha_{k+\frac{1}{2}}< \alpha_{k}. $$

*Furthermore*, $\alpha_{k+\frac{1}{2}}\leqslant c \alpha_{k}$ *if there exists a number* *c* ($0< c<1$) *that satisfies*

$$\bigl\Vert P_{\bar{\varOmega}}(Y_{k+\frac{1}{2}}-W_{k+\frac{1}{2}}) \bigr\Vert _{F}\geqslant (1-c) \Vert Y_{k+\frac{1}{2}}-W_{k+\frac{1}{2}} \Vert _{F}. $$

### Proof

From Method 2.3, we can see that

$$\begin{aligned} \alpha_{k+\frac{1}{2}}&\leqslant \bigl\Vert \operatorname{vec}(D)- \operatorname{vec}\bigl(P_{\varOmega}(W_{k+\frac{1}{2}})\bigr) \bigr\Vert _{F} = \bigl\Vert \operatorname{vec}\bigl(P_{\varOmega}(Y_{k+\frac{1}{2}}) \bigr)-\operatorname{vec}\bigl(P_{\varOmega}(W_{k+\frac{1}{2}})\bigr) \bigr\Vert _{F} \\ &= \Vert Y_{k+\frac{1}{2}}-W_{k+\frac{1}{2}} \Vert _{F}- \bigl\Vert P_{\bar{\varOmega }}(Y_{k+\frac{1}{2}}-W_{k+\frac{1}{2}}) \bigr\Vert _{F}. \end{aligned}$$

When

$$\bigl\Vert P_{\bar{\varOmega}}(Y_{k+\frac{1}{2}}-W_{k+\frac{1}{2}}) \bigr\Vert _{F}\geqslant (1-c) \Vert Y_{k+\frac{1}{2}}-W_{k+\frac{1}{2}} \Vert _{F}, $$

we have

$$\begin{aligned} \alpha_{k+\frac{1}{2}}&\leqslant c \Vert Y_{k+\frac{1}{2}}-W_{k+\frac {1}{2}} \Vert _{F} \leqslant c \Vert Y_{k+\frac{1}{2}}-W_{k} \Vert _{F} \\ &=c \bigl\Vert P_{\varOmega}(Y_{k})-P_{\varOmega}(W_{k}) \bigr\Vert _{F} =c \bigl\Vert \operatorname{vec}(D)-\operatorname{vec}\bigl(P_{\varOmega}(W_{k}) \bigr) \bigr\Vert _{F} =c \alpha_{k}. \end{aligned}$$

If $c=1$,

$$\bigl\Vert P_{\bar{\varOmega}}(Y_{k+\frac{1}{2}}-W_{k+\frac{1}{2}}) \bigr\Vert _{F}\geqslant0 $$

holds true.

Thus,

$$\alpha_{k+\frac{1}{2}}< \alpha_{k} $$

is true. □

### Lemma 3.3

*Assume that* $\{Y_{k}\}$ *is the feasible matrix sequence generated by Method* 2.3, $\{W_{k}\}$ *is the low*-*dimensional matrix sequence formed by partial singular pairs*, *then*

$$\lim_{k\rightarrow\infty} \Vert Y_{k}-W_{k} \Vert _{F}=0 $$

*if the following conditions are satisfied*:

$$r(W_{k})=r \quad \textit{and}\quad \bigl\Vert P_{\bar{\varOmega}}(Y_{k}-W_{k}) \bigr\Vert _{F}\geq(1-c) \Vert Y_{k}-W_{k} \Vert _{F}. $$

### Proof

From

$$\begin{aligned} \Vert Y_{k}-W_{k} \Vert _{F}&\leq \Vert Y_{k}-W_{k-1} \Vert _{F} \\ &= \bigl\Vert P_{\varOmega}(Y_{k}-W_{k-1}) \bigr\Vert _{F} \\ &\leq c \Vert Y_{k-1}-W_{k-1} \Vert _{F} \\ &\leq\cdots \\ &\leq c^{k} \Vert Y_{0}-W_{0} \Vert _{F}. \end{aligned}$$

Therefore,

$$\lim_{k\rightarrow\infty} \Vert Y_{k}-W_{k} \Vert _{F}=0 $$

holds true. □

### Theorem 3.1

*Assume that there exists a positive number* *c* ($0< c<1$) *such that the feasible matrices* $Y_{k}$ *satisfy the following inequality*:

3.1 $$\begin{aligned} \bigl\Vert P_{\bar{\varOmega}}(Y_{k}-W_{k}) \bigr\Vert _{F}\geq(1-c) \Vert Y_{k}-W_{k} \Vert _{F}, \end{aligned}$$

*then the iteration matrices sequence* $\{Y_{k}\}$ *generated by Method* 2.3 *converges to the optimal solution* $Y^{*}$ *of* (1.2) *when the terminated rule* $\epsilon\rightarrow0$ *is satisfied*.

### Proof

From the Method 2.3, we can see the following:

*Case I*. $\tau_{k+1}=[c_{1}\tau_{k}]$ if

3.2 $$\begin{aligned} \bigl\Vert \operatorname{vec}(D)-\operatorname{vec} \bigl(P_{\varOmega}(Y_{k+1})\bigr) \bigr\Vert _{F}\leq c_{2} \bigl\Vert \operatorname{vec}(D)-\operatorname{vec} \bigl(P_{\varOmega}(Y_{k})\bigr) \bigr\Vert _{F} \end{aligned}$$

holds true. That is,

$$\begin{aligned} \operatorname{dim}(W_{k+1})< \operatorname{dim}(W_{k}), \end{aligned}$$

where $W_{k+1}=U_{k+1}\varSigma_{k+1}V^{T}_{k+1}$.

Therefore, there exists an index $k_{0}$ such that $r(W_{k_{0}})< r(Y^{*})$.

From Lemma 3.1, the inequality (3.1) holds true.

At that time, the procedure can be transferred into Step 4 of Method 2.3, and then $\tau_{k_{0}+1}=\tau_{k_{0}}+1$; repeat it, there exists an index $k_{1}$ such that $r(W_{k_{1}})=r(Y^{*})$.

Because of the assumption (3.1) and Lemma 3.2,

$$\begin{aligned} \lim_{\alpha_{k}\rightarrow0} \bigl\Vert D-P_{\varOmega}(Y_{k}) \bigr\Vert _{F}=0 \end{aligned}$$

is true under the restricted condition $r(W_{k})=r(Y^{*})$, $k>k_{1}$.

From Lemma 3.3, we have

$$\begin{aligned} \lim_{k\rightarrow\infty} \Vert W_{k}-Y_{k} \Vert _{F}=0. \end{aligned}$$

Hence,

$$\begin{aligned} \lim_{k\rightarrow\infty}Y_{k}= \lim_{k\rightarrow\infty}W_{k}=Y^{*}. \end{aligned}$$

*Case II*. We assume that there exists an index $k_{2}$ such that the inequality (3.1) holds false but $r(W_{k_{2}})>r(Y^{*})$, and then the procedure can be transferred into the Step 4 of Method 2.3. Because of the assumption (3.1) and Lemma 3.2, we know that there exists an index $k_{3}$ such that the following holds true:

$$\begin{aligned} \alpha_{k_{3}+\frac{1}{2}}< \min\{\alpha_{1},\alpha_{2},\ldots, \alpha _{k_{3}}\}. \end{aligned}$$

At that time, $\tau_{k_{3}+1}=\tau_{k_{3}}-1$, say, the number of dimensionality is decreasing. Repeat the above again and again until there exists an index $k_{4}$ such that $r(W_{k_{4}})=r(Y^{*})$.

That is, we always have the following:

$$\begin{aligned} \lim_{k\rightarrow\infty}Y_{k}=\lim_{k\rightarrow\infty}W_{k}=Y^{*}. \end{aligned}$$

The theorem has been proved. □

## Numerical experiments

It is well known that the OR1MP methd is the most simple and efficient for solving problem (1.1) and the ALM method is one of the most popular and efficient methods for solving problem (1.2). In this section we test several experiments to analyze the performance of our Method 2.3, and compare with the ALM and OR1MP methods.

We compare the methods using general matrix completion problem. In the experiments, $p=m/n^{2}$ denotes the observation ratio, where *m* is the number of observed entries. Here, $p=0.1,0.2,0.3,0.5$ are the different choices of the above ratio. The relative error is $\mathrm{RES}=\frac{\|Y_{k}-D\| _{F}}{\|D\|_{F}}$. The values of the parameters are: $\tau _{0}=100$, $c_{1}=0.8$, $c_{2}=0.9$ and $\epsilon=5e{-}6$.

The results of the experiments are presented in Tables 1–4. From Tables 1–4 we can see that Method 2.3 takes much fewer iterations (denoted by “IT”)) and requires much less computational time (denoted by CPU) than the ALM and OR1MP methods. Thus, Method 2.3 is much more efficient than the other two methods.

**Table 1:** Comparison results of three methods for $p=0.1$

Size	$r(Y_{0})$	Method	RES	IT	CPU
2000 × 2000	20	MAA	6.3989e−05	19	61.0538
		ALM	1.3289e−05	147	814.4656
		OR1MP	1.5920e−02	100	80.2022
3000 × 3000	30	MAA	2.4436e−04	15	113.8439
		ALM	1.2810e−05	155	3448.8579
		OR1MP	1.5641e−02	100	177.3123
4000 × 4000	40	MAA	1.2204e−04	13	163.4071
		ALM	1.1951e−05	166	9876.8939
		OR1MP	1.8042e−02	100	318.5400
5000 × 5000	50	MAA	5.3731e−05	11	210.7015
		ALM	9.6254e−06	173	22,641.7724
		OR1MP	2.0254e−02	100	505.3112

**Table 2:** Comparison results of three methods for $p=0.2$

Size	$r(Y_{0})$	Method	RES	IT	CPU
2000 × 2000	20	MAA	2.4308e−04	10	54.8639
		ALM	9.2238e−06	70	237.4327
		OR1MP	4.3432e−03	100	95.9820
3000 × 3000	30	MAA	5.0593e−05	8	94.3904
		ALM	5.6067e−05	72	863.4068
		OR1MP	5.9270e−02	100	213.9196
4000 × 4000	40	MAA	1.3172e−04	8	166.8769
		ALM	5.4632e−06	72	2336.2629
		OR1MP	8.5351e−03	100	382.8628
5000 × 5000	50	MAA	9.1096e−06	8	248.6944
		ALM	1.0802e−05	64	5141.8507
		OR1MP	1.1188e−02	100	603.1532

**Table 3:** Comparison results of three methods for $p=0.3$

Size	$r(Y_{0})$	Method	RES	IT	CPU
2000 × 2000	20	MAA	6.3561e−05	7	53.9095
		ALM	6.8401e−06	44	74.5572
		OR1MP	2.0841e−03	100	112.3723
3000 × 3000	30	MAA	6.8760e−06	7	112.3313
		ALM	7.8787e−06	46	157.1458
		OR1MP	3.3314e−03	100	251.2893
4000 × 4000	40	MAA	1.4268e−05	6	170.5440
		ALM	8.7585e−06	45	258.4726
		OR1MP	5.5726e−03	100	447.9783
5000 × 5000	50	MAA	1.2876e−05	6	279.8556
		ALM	8.7935e−06	43	420.4459
		OR1MP	8.0070e−03	100	724.6392

**Table 4:** Comparison results of three methods for $p=0.5$

Size	$r(Y_{0})$	Method	RES	IT	CPU
2000 × 2000	20	MAA	1.2636e−05	5	65.7244
		ALM	8.7149e−06	25	44.0239
		OR1MP	7.3980e−04	100	163.9605
3000 × 3000	30	MAA	7.1438e−06	5	149.9019
		ALM	2.7670e−06	24	95.7395
		OR1MP	1.4161e−03	100	338.6274
4000 × 4000	40	MAA	5.7499e−06	5	285.8564
		ALM	3.6098e−06	25	205.6450
		OR1MP	3.1864e−03	100	601.8650
5000 × 5000	50	MAA	5.1190e−06	5	443.8769
		ALM	8.8482e−06	25	245.6650
		OR1MP	4.9806e−03	100	938.3361

**Figure 1:**
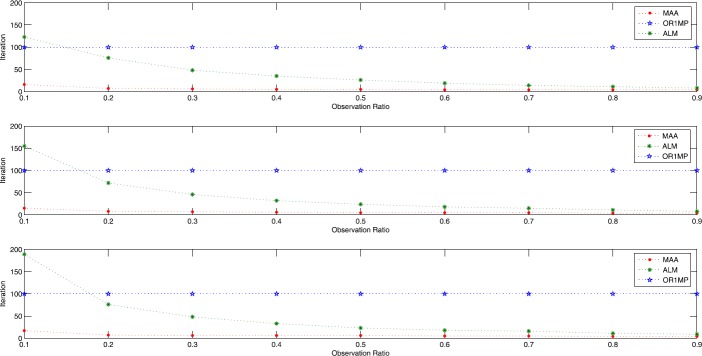
Comparison of completion performance of the MAA, ALM, OR1MP methods with different percentages of observations: the figures correspond to the results on three $3000 \times 3000$ random matrices of rank 10 (top figure), rank 30 (middle figure), rank 50 (bottom figure)

In order to display the effectiveness of our method further, we conduct an experiment on a $3000\times3000$ matrix with three different ranks 10, 30, 50 for three methods with the observation ratios ranging from 0.1 to 0.9, as shown in Fig. 1.

## Concluding remark

Based on the least squares approximation to the known elements, we proposed a manifold-alternative approximating method for the low matrix completion problem. Compared with the ALM and OR1MP methods, shown in Tables 1–4, our method performs better as regards the computing time and the low-rank property. The method can achieve a reduction of the rank of the manifold by gradually reducing the number of the singular value of the thresholding and get the optimal low-rank matrix each iteration step.

## Abbreviations

OMP: orthogonal matching pursuit
OR1MP: orthogonal rank-one matrix pursuit
SVD: singular value decomposition
SVT: singular value theresholding
ASVT: accelerated singular values thresholding method
ALM: augmented Lagrange multiplier
NGP: new gradient projection
RPCA: robust principal component analysis
MAA: manifold-alternative approximating
IT: iteration number
RES: relative errors
CPU: computing time
